# Cancer-associated fibroblasts induce antigen-specific deletion of CD8^**+**^ T Cells to protect tumour cells

**DOI:** 10.1038/s41467-018-03347-0

**Published:** 2018-03-05

**Authors:** Matthew A. Lakins, Ehsan Ghorani, Hafsa Munir, Carla P. Martins, Jacqueline D. Shields

**Affiliations:** Medical Research Council Cancer Unit, University of Cambridge, Cambridge Biomedical Campus, Cambridge, CB2 0XZ UK

## Abstract

Tumours have developed strategies to interfere with most steps required for anti-tumour immune responses. Although many populations contribute to anti-tumour responses, tumour-infiltrating cytotoxic T cells dominate, hence, many suppressive strategies act to inhibit these. Tumour-associated T cells are frequently restricted to stromal zones rather than tumour islands, raising the possibility that the tumour microenvironment, where crosstalk between malignant and “normal” stromal cells exists, may be critical for T cell suppression. We provide evidence of direct interactions between stroma and T cells driving suppression, showing that cancer-associated fibroblasts (CAFs) sample, process and cross-present antigen, killing CD8^+^ T cells in an antigen-specific, antigen-dependent manner via PD-L2 and FASL. Inhibitory ligand expression is observed in CAFs from human tumours, and neutralisation of PD-L2 or FASL reactivates T cell cytotoxic capacity in vitro and in vivo. Thus, CAFs support T cell suppression within the tumour microenvironment by a mechanism dependent on immune checkpoint activation.

## Abstract (web summary)

Tumours employ a variety of strategies to induce immune suppression. Here the authors show that cancer-associated fibroblasts process and cross-present tumour-antigens to T-cells resulting in antigen-specific T cell death and dysfunction via upregulation of both cell death and immune checkpoint signals.

## Introduction

Our immune system is our primary defence mechanism destroying both exogenous and endogenous threats, but tumours have developed strategies to interfere with almost every step necessary for a successful anti-tumour immune response, including mutation of antigen presentation pathways, deregulation of antigen presenting cells, generation of physical barriers and recruitment of suppressive immune subsets, such as Tregs and myeloid derived suppressor cells. Although many immune populations contribute to anti-tumour responses it is the tumour-infiltrating cytotoxic T cells that dominate, their presence correlating with enhanced prognosis^1–3^, and thus many suppressive mechanisms identified act to inhibit T-cell function. With reports of effects on recruitment and behaviour of multiple immune populations, the supporting tumour stroma is emerging as a key source of tumour-promoting inflammation. Moreover, observations that tumour-associated T cells are preferentially found with stromal rich areas of the tumour rather than penetrating into tumour islands^4,5^, introduces the prospect that components of the tumour microenvironment^4,6–11^ may be critical for T cell suppression. Cancer-associated fibroblasts (CAFs), the most abundant stromal population and associated with poor patient prognosis, are emerging as suppressive intermediates within the tumour microenvironment (TME) through secretion of immunomodulatory factors that polarise responsive immune populations, such as macrophages^4,6,8,9,12^. While CD8^+^ T-cell infiltration and cytotoxicity are the most important determinants of anti-tumour immunity^1–3^, it is still unclear as to whether soluble CAF-derived signals are sufficient or able to drive changes in T-cell functional status. Since T cells are often restricted to stromal zones^4,5,13–15^, we sought to determine the mechanisms by which CAFs may mediate dysfunction of CD8^+^ T cells they encounter.

## Results

### CAFs sample and proteolytically process exogenous antigen

**Fig. 1:**
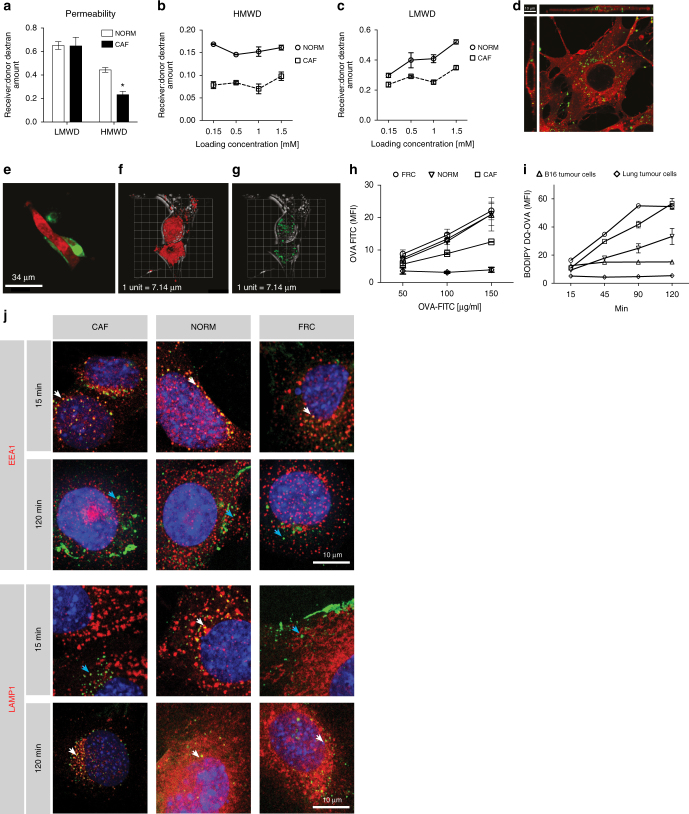
Cancer associated fibroblasts engulf and proteolytically process cellular debris and antigen. **a** Relative permeability of low-molecular weight dextran (LMWD) and high-molecular weight dextran (HMWD) across monolayers of normal (NORM, white bars) or cancer-associated fibroblasts (CAF, black bars). **b**, **c** Apparent permeability across NORM and CAF monolayers of increasing HMWD (**b**) and LMWD concentrations (**c**). **d** Representative confocal micrograph including *z*-plane of HMWD (green) within CAFs (red, Cell Mask). Scale bar, 10 μm. **e** Representative confocal snapshot of CAF (red) interacting with dead and dying tumour cells (green). Twenty-four hours after debris engulfment showing CAF (red, **f**), and intracellular tumour cell debris (green, **g**). **h** Uptake of FITC-labelled OVA by fibroblasts and tumour cells. **i** Processing of DQ-OVA by fibroblasts and tumour cells. **j** Representative micrographs of endosomal compartments (red) showing early endosomes (EEA1) and lysosomes (LAMP1) at 15 and 120 min post ovalbumin pulse (green). Areas of co-localisation (white arrows) and ovalbumin not within labelled compartments (blue arrows) are indicated in each image. Scale bar: 10 μm. Data shown as mean ± SEM. **a** *P < 0.05 vs. NORM fibroblasts (two-tailed unpaired Student’s *t*-test). **h**, **i** ****P < 0.0001 (two-way ANOVA with Tukey post hoc analysis vs. CAFs). NS, not significant. Assays performed in duplicate from three (**h**) or two independent experiments (**i**)

At sites of physiological immune regulation, such as the thymus or lymph node, antigen-specific cell–cell interactions are required to modulate T-cell activity. Antigen-presenting cells (APCs) achieve this through cross-presentation of exogenously sampled and captured antigens upon major histocompatibility complex (MHC)-I, thus we first assessed whether CAFs possess similar capabilities. CAFs isolated from murine lung tumours (Supplementary Fig. 1a–c) were able to generate a physical, size-selective barrier in 2-chamber permeability assays, significantly delaying the transit of large MW material which occurred by both paracellular and transcellular routes, via an active transport process (Fig. 1a–c). Following the observation that large MW dextran was engulfed by CAFs (Fig. 1d), we further established that CAFs scavenged autologous cellular material (Supplementary Fig. 2a) and likewise, debris from dead tumour cells (Fig. 1e, representative snapshot from Supplementary Movie 1) that were directed to discrete intracellular compartments (Fig. 1f, g, representative snapshot from Supplementary Movie 2). To establish the fate of ingested material in a quantitative manner, we utilised the antigen ovalbumin (OVA). While all fibroblast lines and tumour cells derived from lung adenocarcinoma and melanoma engulfed antigen to varying degrees as measured by FITC-OVA (Fig. 1h), DQ-OVA fluorescence confirmed that lymph node fibroblasts (FRCs, which can present antigen and modulate T cells^16^) and CAFs were most efficient at proteolytic processing of intracellular OVA (Fig. 1i and Supplementary Fig. 2b). This was proteasome-independent, instead utilising the endosomal pathway. We noted that CAFs exhibited delayed antigen processing kinetics compared to FRCs and normal fibroblasts (Fig. 1i, j and Supplementary Fig. 3a). The initial processing delay recorded in CAFs was highly reminiscent of professional APCs, where retention of antigen within early endosomes enhances cross-presentation to cognate antigen-specific T cells^17–19^.

### CAFs cross-present exogenous antigen

**Fig. 2:**
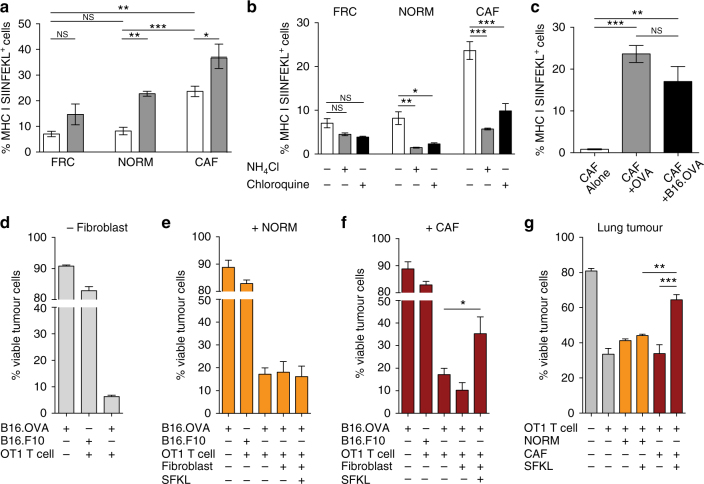
CAF cross-presentation of processed antigen protects tumour cells from T cell killing. **a** Quantification of MHC I SIINFEKL detected in FRCs, CAFs and normal fibroblasts following OVA pulse (white bars), and when pulsed in tumour conditioned media (grey bars). **b** MHC I SIINFEKL detection in the presence of ammonium chloride (grey bars), chloroquine (black bars) and vehicle control (white bars). **c** MHC I SIINFEKL detected on CAFs after co culture with B16.OVA tumour cells, or CAFs pulsed with soluble OVA. **d** T cell killing of control parental (B16.F10) and antigen bearing (B16.OVA) target tumour cells in the absence of fibroblasts. **e**, **f** B16 viability when introduced to OT-I T cells previously conditioned in the presence/absence of normal fibroblasts (**e**), CAFs (**f**) and presence/absence of antigen as indicated on graphs. **g** Lung tumour cell viability when introduced to OT-I T cells previously conditioned in the presence/absence of normal fibroblasts (yellow bars), CAFs (red bars) and presence/absence of antigen as indicated on graphs. Data shown as mean ± SEM. *p < 0.05, **p < 0.01 and ***p < 0.001, one-way ANOVA with Tukey post hoc analysis. NS, not significant. Assays performed in triplicate (**a**,** c**,** g**) or duplicate (**b**) from two experiments or (**d**-**f**) three experiments. Comparisons indicated by horizontal lines

Indeed, in CAFs, delayed endosome-mediated processing translated to enhanced cross-presentation of OVA_257–264_ peptides complexed with MHC-I compared to either normal fibroblasts or FRCs (detected with monoclonal antibody 25-D1.16, Fig. 2a and Supplementary Fig. 3b). Cross presentation could be inhibited with by chloroquine or ammonium chloride, which blocked endosomal processing pathways (Fig. 2b). Using OVA-expressing and GFP-tagged tumour cells as a source of material we further confirmed the capacity of CAFs to sample, process and cross-present tumour-derived antigen consistent with intracellular events observed for soluble antigen (Fig. 2c and Supplementary Fig. 3c).

### CAF-conditioned T cells exhibit reduced cytotoxic capacity

We next determined whether CAF-driven antigen cross-presentation was able to negatively regulate T cells, translating to a tumour cell survival advantage. We performed triple culture assays incorporating CAFs, T cells and tumour cells where tumour cell fate could be recorded, and in which we could simultaneously monitor T cell and CAF functional status and activity. Normal fibroblasts, irrespective of whether they were pre-exposed to OVA or not, did not confer protection to OVA-expressing target tumour cells as fibroblast-conditioned OT-I T cells still efficiently killed their targets (Fig. 2d, e). In contrast, tumour cell survival was significantly enhanced when OT-I T cells were conditioned in the presence of CAFs prior to culture with tumour cells (Fig. 2d, f). This CAF-driven suppression of OT-I T cell cytotoxicity was antigen-specific and antigen-dependent since the enhancement in tumour cell viability was only observed when CAFs were previously OVA-exposed (Fig. 2d-f). Moreover, when OTI T cells were removed from the influence of CAFs, enhanced tumour cell survival was maintained, supporting the hypothesis that direct CAF-T-cell interactions drive non-reversible functional modifications rather than reliance on a long term, CAF-derived, soluble stimulus (Supplementary Fig. 4a). Equivalent preservation of tumour cell viability was also measured when tumour cells themselves, instead of OVA, were used as the source of antigenic material used by CAFs to condition T cells (Supplementary Fig. 4b). The protective effects measured were antigen rather than tumour dependent as similar protection was also observed in OVA-expressing target lung cancer cells (Fig. 2g and Supplementary Fig. 4b).

### CAFs induce T cell death via PD-L2 and FASL engagement

**Fig. 3:**
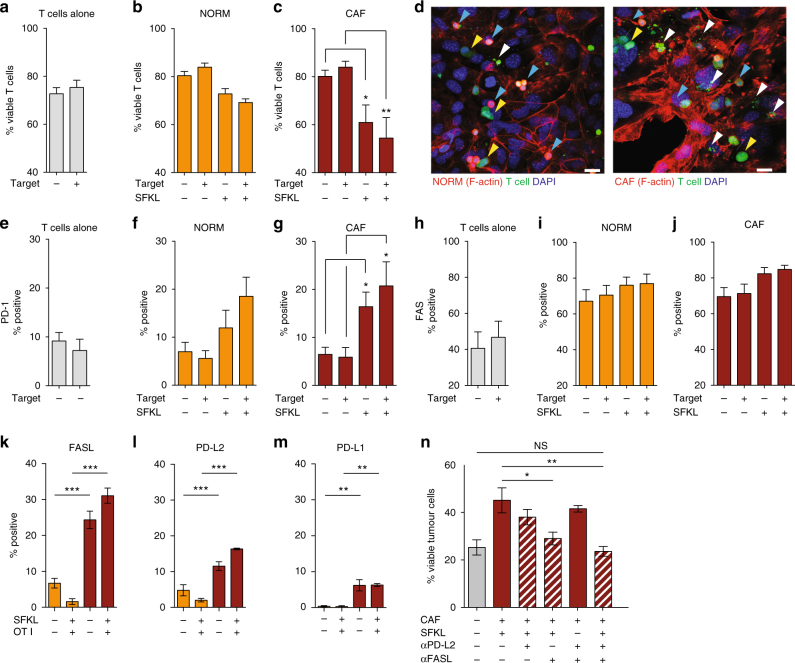
CAFs reduce CD8^+^ T cell viability in an antigen-specific death ligand dependent manner. **a-c** Quantification of T cell viability after conditioning in culture (**a**) or in the presence of normal fibroblasts (**b**), CAFs (**c**) and presence/absence of antigen and target tumour cells as indicated on graphs. **d** Representative micrographs of T cells (green) co-cultured with normal fibroblasts (red, f-actin) and CAF (red, f-actin) showing intact T cells (yellow arrows), T cells surrounded by actin bundles (blue arrows), and fragmented T cells (white arrows). Nuclei stained with DAPI (blue). Quantification of PD-1 expression (**e**-**g**) and FAS expression (**h**-**j**) on T cells following culture with normal fibroblasts (**f**, **i**), CAFs (**g**, **j**). **k**-**m** Quantification of cognate ligand expression by co-cultured fibroblasts by flow cytometry; FASL (**k**), PD-L2 (**l**) and PD-L1 (**m**) expression by NORM (yellow bars) and CAFs (red bars) with and without antigen and antigen-specific OT-I T cells as indicated. **n** B16.OVA tumour cell viability following culture with CAFs, antigen and OT-1 T cells following CAF-specific neutralisation of PD-L2 and/or FASL. Data shown as mean ± SEM. *p < 0.05, **p < 0.01, ***p < 0.001, one-way ANOVA with Tukey’s or Dunnett’s post hoc analysis. NS, not significant. Comparisons indicated by horizontal lines. Scale bar 40 μm. Assays performed in or quadruplet from two experiments (**a**-**c**) or duplicate from three (**e**-**m**) or two (n) independent experiments

As the capacity for antigen-specific T cells to kill their target tumour cells was dramatically impaired when conditioned by antigen-loaded CAFs, we looked to identify the mechanism of CAF-mediated T cell suppression. Comparisons of OT-I T cell viability following culture alone, with normal fibroblasts or CAFs revealed that the CAFs caused an antigen-dependent decrease in OT-I T cell viability (Fig. 3a-c). T cell death was mediated through the expression and engagement of immune checkpoints FAS and PD-1 (Fig. 3e-j). While FAS expression increased upon exposure to stromal cells (Fig. 3h-j), stimulation of PD-1 was dependent upon the presence of antigen (Fig. 3e-g). Reciprocal expression of ligands FASL and PD-L2 was detected to a greater extent on CAFs than normal fibroblasts (Fig. 3k, l). Surprisingly, PD-L2 rather than PD-L1 was the dominant PD-1 ligand (Fig. 3l, m). Although, PD-L2 physiologically displays a more restricted pattern of expression limited to APCs^20–22^, functional expression has been reported on human colonic^23^, lung^24^ and melanoma fibroblasts^25^. Moreover, flow cytometric analysis confirmed that non-deleted T cells also upregulated the exhaustion marker LAG3 (Supplementary Fig. 4c), and when PD-L2 or FASL were blocked specifically on CAFs using neutralising antibodies, the capacity of OT-I T cells to kill their targets was restored (Fig. 3n). These data imply CAFs utilise tumour antigen cross-presentation and coincident upregulation of key immune checkpoint ligands to protect tumour cells from immune destruction by driving antigen-specific T cell death, and functional impairment of the remaining cytotoxic T-cell compartment.

### PD-L2 and FASL inhibition impair CAF-driven T cell suppression

**Fig. 4:**
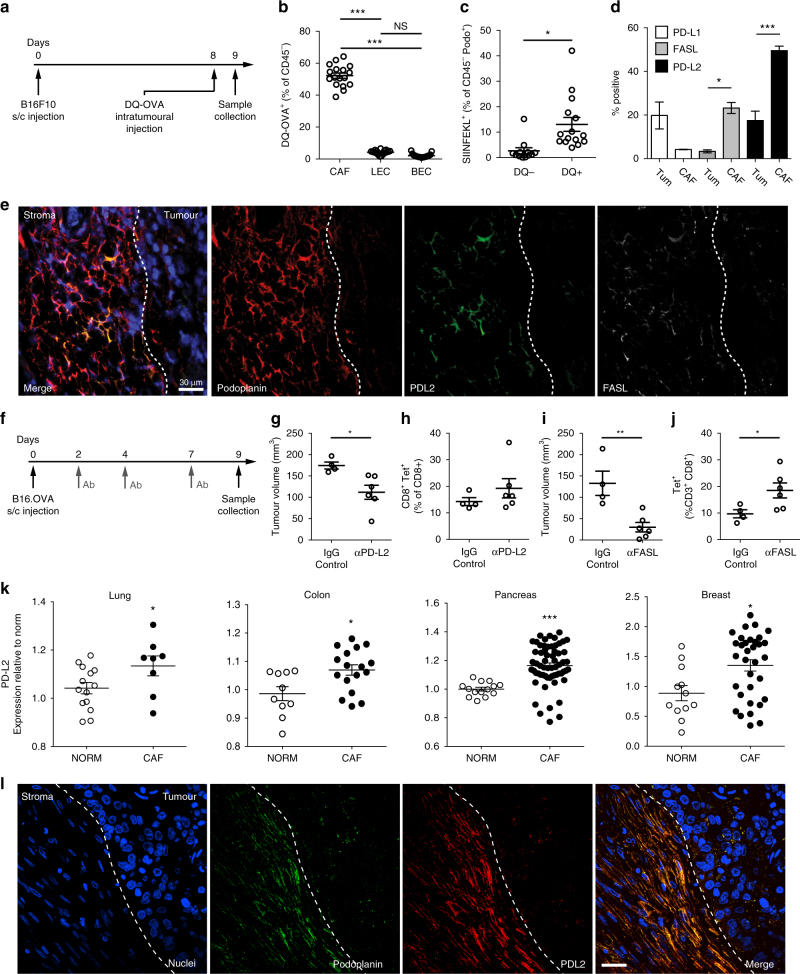
CAFs process and present model antigen and kill antigen-restricted T cells via PD-L1/2 and FASL. **a** Schematic of in vivo experimental design. Following intratumoural injection of DQ-OVA, tumours and lymph nodes were collected for analysis. **b** DQ-OVA processing by CAFs, LECs or BECs as a percentage of live CD45^−^ cells. **c** MHCI-SIINFEKL expression by DQ-OVA-negative CAFs compared with DQ-OVA-positive CAFs. **d** In vivo PD-L1 (white bars), FASL (grey bars) and PD-L2 (black bars) expression by tumour cells (Tum) and CAFs (CAF). **e** Representative confocal micrograph of B16.OVA tumour for podoplanin (CAFs, red), PD-L2 (green) and FASL (white). Scale bar: 30 μm. **f** Schematic of in vivo blocking antibody experiments. **g** Day 9 tumour volumes after PD-L2 neutralisation. **h** MHCI-SIINFEKL pentamer-specific CD8^+^ T cell frequency within control and treated tumours. **i** Day 9 tumour volumes after FASL neutralisation. **j** MHCI-SIINFEKL pentamer-specific CD8^+^ T cell frequency within control and treated tumours. **k** Publically available microarrays analysed for PD-L2 in normal fibroblasts and CAFs from human lung (GSE22862), colon (GSE46824 and GSE1257), pancreatic (GSE21440) and breast cancers (GSE29270). **l** Representative image of tumour-stroma interface of stage III lung adenocarcinoma from tissue microarray; CAFs (podoplanin, red), PD-L2 (green), nuclei (DAPI, blue). Dashed line: tumour border. Demonstrable staining was not detected in normal lung tissues. Scale bar: 50 μm. **b**, **d** Data shown as mean ± SEM. *P < 0.05, ***P < 0.001 (one-way ANOVA with Tukey’s or Dunnett’s post hoc analysis). NS, not significant. **b** *n* = 18 tumours from three experiments. **c** Data shown as mean ± SEM. *P < 0.05. *n* = 12–15 tumours from two experiments. **g**–**k** Data shown as mean ± SEM. *P < 0.05, **p < 0.01. (two-tailed unpaired Student’s *t*-test). *n* = 4 and six tumours from two experiments. Symbols represent individual tumours (**b**,** c**,** g**–**j**) or humans sample (**k**)

Antigen processing and presentation by CAFs were also detected in tumours of immune competent mice (Fig. 4a–c), as were preferential expression and localisation of PD-L2 and FASL to the CAF compartment (Fig. 4d, e) validating in vitro data. This corresponded with reduced numbers of OTI T cells in antigen-bearing tumours (Supplementary Fig. 5a–g) and upregulated FAS and PD-1 expression exclusively on remaining intratumoural, antigen-specific T cells (Supplementary Fig. 5h–k). Significantly, systemic neutralisation of PD-L2 or FASL activity supported decreases in tumour volume (Fig. 4g, i) and coincident enhanced infiltration of antigen-specific CD8^+^ T cells (Fig. 4h, j) in the absence of effects in immune or stromal compartments (Supplementary Fig. 6a–i). To confirm a role for CAFs in mediating these effects in vivo, we induced B16.OVA tumours through injection of tumour cells alone or in combination with CAFs at a 1:1 ratio (Supplementary Fig. 7a, b verifying presence of CAFs in vivo after co-injection). Specifically, we utilised *gld/gld* mice, homozygous for the *fasl*^*gld*^ mutation and lacking functional FASL expression. Tumours in *gld/gld* mice were smaller and contained significantly more antigen specific CD8^+^ T cells than the mixed tumours in which CAFs were the only source of FASL or WT C57 mice (Supplementary Fig. 7c–e). The mixed tumours in *gld/gld* mice were indeed comparable with tumours in wild-type C57 mice (Supplementary Fig. 7c–e) where other cell populations may be an additional source of FASL, supporting a specific role for CAF-mediated FASL in T cell deletion observed both in vivo and in vitro (Fig. 3). We next compared RNA expression of inhibitory ligands in publically available microarray data sets for CAFs from human tumour tissues and normal counterparts, and confirmed elevated PDCD1LG2 gene expression in human lung cancer (Fig. 4k). This observation could be extended to CAFs of colon, pancreatic, and breast cancers (Fig. 4k), but not in prostate cancer. Consistent with in vitro and in vivo findings of PD-L2 as the predominant CAF-expressed immune-inhibitory ligand, no significant change in CD274 was apparent in the human CAF transcriptomes examined (Supplementary Fig. 8a). Moreover, lung and colon cancers exhibited enrichment of FASLG (Supplementary Fig. 8b), but a greater degree of variability was observed between tumour types, with no increase noted in pancreatic or breast data sets examined. PD-L2 enrichment within stromal regions of lung tumours was then confirmed at the protein level by immunofluorescence (Fig. 4l). Together, these mirrored the compartment-specific expression patterns detected in murine models, which when functionally blocked translated to enhanced intratumoural antigen-specific cytotoxic T-cell function.

## Discussion

As they progress, tumours develop strategies to interfere with effective anti-tumour immune responses, ranging from the recruitment of suppressive immune populations to the deletion or functional impairment of tumour-reactive T cells. Recent paradigm shifting immunotherapy platforms that release endogenous anti-tumour immune responses from inhibition have shown remarkable success in some cancers, but the majority of patients do not respond or attain long-lasting benefit^26–30^. A potential reason underlying the varying therapeutic responses is the impact of the tumour microenvironment itself upon immune repertoires and functional status. Thus, increasing our understanding of tumour-associated suppressive networks will be necessary to improve the efficacy and development of immune based therapeutics. We demonstrate a new biological function for fibroblasts in a tumour, showing that CAFs directly contribute to the suppression of anti-tumour T-cell responses by adopting characteristics reminiscent of antigen presenting cells. This mechanism has two key features: first, fibroblasts sample, process and cross-present antigen complexed with MHC I. Second, co-incident antigen-specific upregulation of FAS/FASL and PD-1/PD-L2 on T cells and CAFs respectively, drives the death and dysfunction of tumour specific T cells resulting in enhanced tumour viability. Together with human data demonstrating PD-L2 and FASL enrichment within stromal zones of lung tumours, this CAF-mediated mechanism reveals new insight into the cell biology of tumour-associated fibroblasts; helping to explain why cancer associated fibroblasts are associated with poor patient prognosis, and illustrating a novel mechanism of T cell depletion and dysfunction within tumours.

## Methods

### Animal use

All experiments involving animals were performed in accordance with UK Home Office regulations, PPLs 80/2574 and P88378575. For mouse models, G*Power was used to estimate samples sizes required to achieve 80% power with 5% threshold. For syngeneic tumours, 2.5 × 10^5^ B16.F10 cells (or variants) were inoculated subcutaneously into the shoulders of 8–9-week-old immune competent female C57BL/6 mice. Animals were excluded only if tumours failed to form or if health concerns were reported. Tumour size was monitored using the ellipsoid formula (length × width^2^)/2. For experiments in which in vivo antigen processing was measured, 10 μl of 5 mg ml^−1^ DQ OVA was injected intratumourally. Twenty-four hours later, mice were killed and tumours were collected for analysis. For blocking studies, animals were assigned to control or tumour groups randomly and B16.OVA bearing mice received neutralising antibodies against PD-L2 (clone TY25, 200 μg per mouse) or FASL (clone MFL4, 200 μg per mouse, both BioLegend) or matched concentration normal IgG controls every 2 days via intra-peritoneal administration. Tecnhicians performing treatments were blinded to reagents. Tumours were collected for flow cytometry analysis of immune compartments and antigen specific T cells using pentamers against SIINFEKL (ProImmune). Mice deficient for FASL (B6Smn.C3-*Fasl*^*gld*^/J, Jax Lab), referred to as *gld* mice in text, and wt C57BL/6 mice were injected with either 2.5 × 10^5^ B16.F10 cells or 1:1 mix of B16.F10 cells:CAF and tumour size monitored. Tumours were collected for flow cytometry analysis of immune compartments and antigen specific T cells using pentamers against SIINFEKL (ProImmune). C57BL/6-Tg(CAG-EGFP)1Osb/J GFP mice (Jax Lab) were used to confirm the presence of CFSE far red-labelled (C34564, Molecular Probes) CAFs in mixed tumours. At day 9, tumours were collected and prepared for flow cytometry analysis and confocal imaging. For adoptive transfer assays, splenocytes were isolated from either OT-I or C57BL/6 mice. Briefly, tissues were mechanically disrupted prior to red blood cell lysis using ammonium chloride lysis buffer (150 mM NH_4_Cl, 10 mM NaHCO_3_, pH 7.4, 0.4% EDTA). Splenocytes were activated with 10 nM SIINFEKL peptide (OT-I) or CD3/CD28 (C57BL/6) for 2 days. Antigen specific or wild-type CD8^+^ T cells were isolated using a MACs CD8^+^ T Cell Isolation Kit (Miltenyi Biotec) and expanded in IL-2 (20 ng ml^−1^) containing media for a further 5 days. Expanded CD8^+^ T cells were then live-labelled with cell trace CFSE green or far red respectively. Seven days after B16.OVA or B16.F10 induction 5 × 10^6^ green and 5 × 10^6^ for red T cells were injected intravenously. For early time points, tumours, draining lymph nodes and spleens were collected after 18 h and processed for flow cytometric analysis. Later time points, 72 h after adoptive transfer, were collected and treated as for the early time point.

### Cell isolation

CAFs were isolated from primary lung tumours derived from LSL-Kras^G12D/+^;p53^LSL-R270H/ER^ tumour-bearing mice^31,32^ 21 weeks after adenoviral Cre infection (5 × 10^6^ pfu mouse^−1^)^33^. Tissues were digested (collagenase/dispase) and seeded into 6 well plates. A differential adhesion protocol was used to select for tumour cells vs. CAFs and purity of tumour:CAFs culture confirmed by FACS (EpCam:Pdgfrα). Unlike the accompanying tumour cells, CAFs did not undergo Cre-mediated recombination and were therefore functionally wild type for Kras (hemizygous) and p53-null. Normal lung fibroblasts were isolated from control, non-adenoviral treated mice (p53^LSL-R270H/ER^). Melanomas and corresponding fibroblasts were isolated from B16.F10 inoculated into cagEGFP.BL6 mice and characterised as described below. Tissues were digested as described for tissue processing and stained for CD45, CD31 and podoplanin prior to FACs sorting. Tumour cells were excluded by GFP-negative status and immune, endothelial and pericyte compartments were also excluded. Remaining cells were plated for culture and characterisation. FRCs were isolated from murine lymph nodes^16,32,34^. Briefly, pooled lymph nodes were mechanically disrupted and digested in a 500 μl mixture of 1 mg ml^−1^ collagenase A (Roche) and 0.4 mg ml^−1^ DNase I (Roche) in PBS at 37 °C for 30 min with 600 r.p.m. rotation. Following centrifugation at 1000 r.p.m. for 5 min, the supernatant was discarded and replaced with 500 μl of PBS containing 1 mg ml^−1^ Collagenase D (Roche) and 0.4 mg ml^−1^ DNase I. The mixture returned to 37 °C for 20 min with 600 r.p.m. rotation before addition of EDTA (final concentration 10 mM). Suspensions were passed through a 70 μm mesh before plating into 6 well plates. Cultures were expanded and tested for purity by flow cytometry. At passage 2, any contaminating endothelial cells were removed by FACS sorting.

### Cell culture

B16.F10 and B16.OVA cells (CRL-6475, ATCC) were maintained in DMEM with 10% fetal bovine serum (both Life Technologies) and 1% penicillin-streptomycin (Sigma-Aldrich). Lung tumour cells were maintained in HAMS-F12 with 10% fetal bovine serum (both Life Technologies) and 1% penicillin-streptomycin (Sigma-Aldrich). FRCs were maintained in RPMI with 10% fetal bovine serum, 10 mM HEPES (all Life Technologies), 1% penicillin-streptomycin, 15 μM β-mercaptoethanol (both Sigma-Aldrich). CAFs and normal fibroblasts were maintained in DMEM with 1.5 g L^−1^ NaHCO_3_, 10% fetal bovine serum (both Life Technologies) and 1% penicillin-streptomycin (Sigma-Aldrich). T cells were maintained in IMDM with 5% fetal bovine serum (both Life Technologies), 1% pencillin-streptomycin and 15 μM β-mercaptoethanol (both Sigma-Aldrich). All cells in culture were routinely tested for mycoplasma contamination (MycoAlert Detection Kit, Lonza).

### Tumour tissue processing for flow cytometry

Tumours were mechanically disaggregated and allowed to digest in a 1 ml mixture of 1 mg ml^-1^ collagenase A (Roche) and 0.4 mg ml^−1^ DNase I in PBS at 37 °C for 1 h with 600 r.p.m. rotation. The mixture was gently pipetted up and down every 10–20 min. The mix was then refreshed with 1 ml PBS containing 1 mg ml^−1^ Collagenase D (Roche) and 0.4 mg ml^−1^ DNase I and then returned to 37 °C for 1 h with 600 r.p.m. rotation. Collagenase was then neutralised with EDTA (final concentration 10 mM) and cells were passed through a 70 μm mesh prior to immunostaining. Single-cell suspensions were stained with fixable viability dye eFluor 780 (eBioscience) or live/dead violet (Molecular Probes) and combinations of the following fluorescently conjugated antibodies; SIINFEKL pentamer (ProImmune), podoplanin (clone 8.1.1), CD31 (clone 390), CD4 (clone GK1.5), CD8a (clone 53.6–7), CD45 (clone 30-F11), FAS (clone 15A7), FASL (clone MFL3), Interferon-gamma (clone XMG1.2), PD-1 (clone RMP1-30), PD-L1 (clone 10F-9G2), PD-L2 (clone TY25), LAG3 (clone C9B7W), Tim3 (clone B8.2C12, all 1:300, all BioLegend). Flow cytometry was performed on CyAn ADP (Beckman Coulter) and LSR Fortessa (BD Biosciences) analysers. Unstained, viability dye only, and single-stained compensation beads (Invitrogen) served as controls. Doublets were gated out using forward-scatter width/height and sideward- scatter width/height event characteristics. Offline analysis was carried out on FlowJo (Treestar).

### CAF characterisation

FACs analysis of fibroblasts was performed using a panel of typical CAF markers; podoplanin (clone 8.1.1), PDGFRα (clone APA5) and β (clone APB5) (all 1:300, all BioLegend), FAP-α (1:50, AF3715; R&D Systems); and markers to exclude immune cells (MHCII; clone KH74 and CD45; clone 30-F11), epithelial cells (EpCAM, clone G8.8; Biolegend) and endothelial cells (CD31, clone 390; BioLegend). Morphological characteristics assed and visualised using an EVOS microscope and functional analysis was measured in terms of collagen gel contraction capacity. 1.5 × 10^5^ cells were seeded into 2 mg ml^−1^ collagen gel (BD Biosciences) and detached from the sides of 24 well plates. Gel contraction was imaged over time and quantified as percentage area change over time. CAFs displayed typical CAF morphology, marker profiles and functionality were utilised (Supplementary Fig. 1).

### In vitro ovalbumin uptake and processing

For antigen uptake analysis, cells (3 × 10^4^ per well in a 24 well plate) were incubated with 50, 100 or 150 μg ml^−1^ FITC-OVA in complete media for 15 min before being washed with ice-cold PBS + 5% FBS. For antigen processing, 3 × 10^4^ cells per well were seeded in 24 well plates and pulsed with DQ-OVA, 100 μg ml^−1^ in PBS for a 15 min pulse. Wells were then washed with ice-cold PBS + 5% FBS and cultured in pre-warmed full culture media for 0, 15, 45, 90, or 120 min at 37 °C. Uptake of FITC-OVA and processing of DQ-OVA were detected on a CyAn^TM^ ADP Analyser (Beckman Coulter).

### In vitro inhibition of ovalbumin uptake and processing

To determine the route of antigen processing and impact of this route on cross presentation, wells were incubated in the presence of lactacystin (0.02 μM), ammonium chloride (0.5 μM), MG132 (0.01 μM) or chloroquine (0.05 μM) for 1 h before, and throughout incubation with antigen.

### Immunofluorescence

Collected tumours were embedded in OCT medium (TissueTek). 10 μm sections were fixed in ice-cold acetone for 10 min, blocked with 5% chicken serum in PBS, and incubated with the following primary antibodies, 4 °C overnight: goat anti-podoplanin (BAF3244, 1:50, R&D Systems), PE conjugated anti-PDL2 (107206, 1:100, BioLegend), APC-conjugated anti-FASL (17–5911, 1:100, eBiosciences), Hamster anti-CD3e (553238, 1:100, BD Pharmingen), Biotinylated-rat anti-Thy1 (ab25285, 1:100, Abcam). Cell nuclei were counterstained with DAPI and sections were mounted in ProLong Gold (Invitrogen). Confocal images were taken using Leica SP5 confocal microscope and processed with Volocity (Perkin Elmer). For immunocytochemistry, cells were fixed following in vitro assays and stained for Rab5 (C8B1), Rab7 (D95F2), EEA1 (2411S) and LAMP1 (1D4B), all Cell Signalling Technologies, all 1:100). Cell nuclei were counterstained with DAPI and sections were mounted in ProLong Gold (Invitrogen). Confocal images were taken using Leica SP5 confocal microscope and processed with Volocity (Perkin Elmer). For human tissues, lung TMAs were purchased from Cambridge Biosciences. Slides were dewaxed in xylene and rehydrated in graded alcohols prior to antigen retrieval in sodium citrate pH6. A non-immune block was performed for 1 h prior to incubation in primary antibodies at 4 °C overnight: sheep anti-human podoplanin (AF3670, 1:20), mouse anti-human PD-L2 (MAB1224, 1:80, both R&D Systems). Samples were washed and incubated in fluorescently conjugated secondary antibodies before cell nuclei were counterstained with DAPI and sections were mounted in ProLong Gold (Invitrogen) ready for imaging.

### Live imaging and post analysis

To live label cultures, Cell Tracker Stains CMTPX (C34552) or CMFDA (C2925) (both Life Technologies) were employed and used according to manufacturer’s guidelines. To induce tumour cell death for debris uptake assays, GFP-labelled tumour cells were killed with puromycin (200 μg ml^−1^) prior to incubation with labelled CAFs. To determine the intracellular compartment of engulfed tumour debris, cells were incubated with LysoTracker (Life Technologies) according to manufacturer’s guidelines. Untreated or live-labelled cells were imaged over a period of 24 h on a Leica DMi8 microscope with environmental chamber. Images were taken every 10 min. After live imaging, cells were fixed in 4% PFA for 10 min at RT and stained. Actin cytoskeleton was visualised with Phalloidin-Atto 647N (Sigma-Aldrich).

### T cell/CAF phenotyping and co/tri-cultures and phenotyping

10^4^ fibroblasts were seeded in to wells of a 24 well plate in control media or media containing 5 nM SIINFEKL peptide. Cells were incubated overnight and washed thoroughly three times with PBS + 5% FBS. 10^5^ antigen-specific or wild-type T cells were co-cultured with fibroblasts, or alone, for 48 h. Baseline T cell and fibroblast flow cytometric phenotyping was carried out at this point. T cells were collected and remaining fibroblasts were collected by cell dissociation and both cell types were stained for phenotypic markers and analysed by flow cytometry. For T cell phenotyping combined with target cells, antigen bearing tumour cells were stained with cell tracker green (Life Technologies, CFDA) prior to co-culture. Three hours after target tumour cell addition, brefeldin A (BFA, BioLegend, 1:1000) was added to the cells and T cell phenotype was assessed after a further 3 h BFA treatment. For tumour cell killing assay, tumour cell viability was assessed by live/dead staining (Life Technologies) and Annexin V (BioLegend) staining after overnight incubation.

### Statistical analyses

Statistical analyses were performed using GraphPad Prism 6 software (GraphPad). For comparisons of three or more groups, data were subjected to one-way ANOVA analysis, followed by Dunnett’s multiple comparisons test when comparing every mean to a control mean, or Tukey’s multiple comparisons test when comparing every mean to every other mean. When two groups were compared, a two-tailed paired or unpaired Student’s *t*-test was applied. Data are represented as mean ± SEM, and *p* ≤ 0.05 was considered significant.

### Data availability

The expression of PD-L2 by human fibroblasts from normal and tumour tissues referenced during the study; lung (GSE22862), breast (GSE29270), pancreatic (GSE21440) and colon (GSE46824 and GSE1257) are available in a public repository from the NCBI GEO2R website (http://www.ncbi.nlm.nih.gov/geo/geo2r/).

All the other data supporting the findings of this study are available within the article and its Supplementary Information files and/or from the corresponding author upon reasonable request.

## Electronic supplementary material

Supplementary Information

Peer Review File

Description of Additional Supplementary Files

Supplementary Movie 1

Supplementary Movie 2
