# Supplementary material for: Cancer-associated fibroblasts induce antigen-specific deletion of CD8+ T Cells to protect tumour cells
Source: Nat Commun. 2018 Mar 5;9:948. doi: 10.1038/s41467-018-03347-0 (PMC5838096; doi:10.1038/s41467-018-03347-0)
Supplement: Supplementary file 1 — Supplementary Information [file 41467_2018_3347_MOESM1_ESM.pdf]

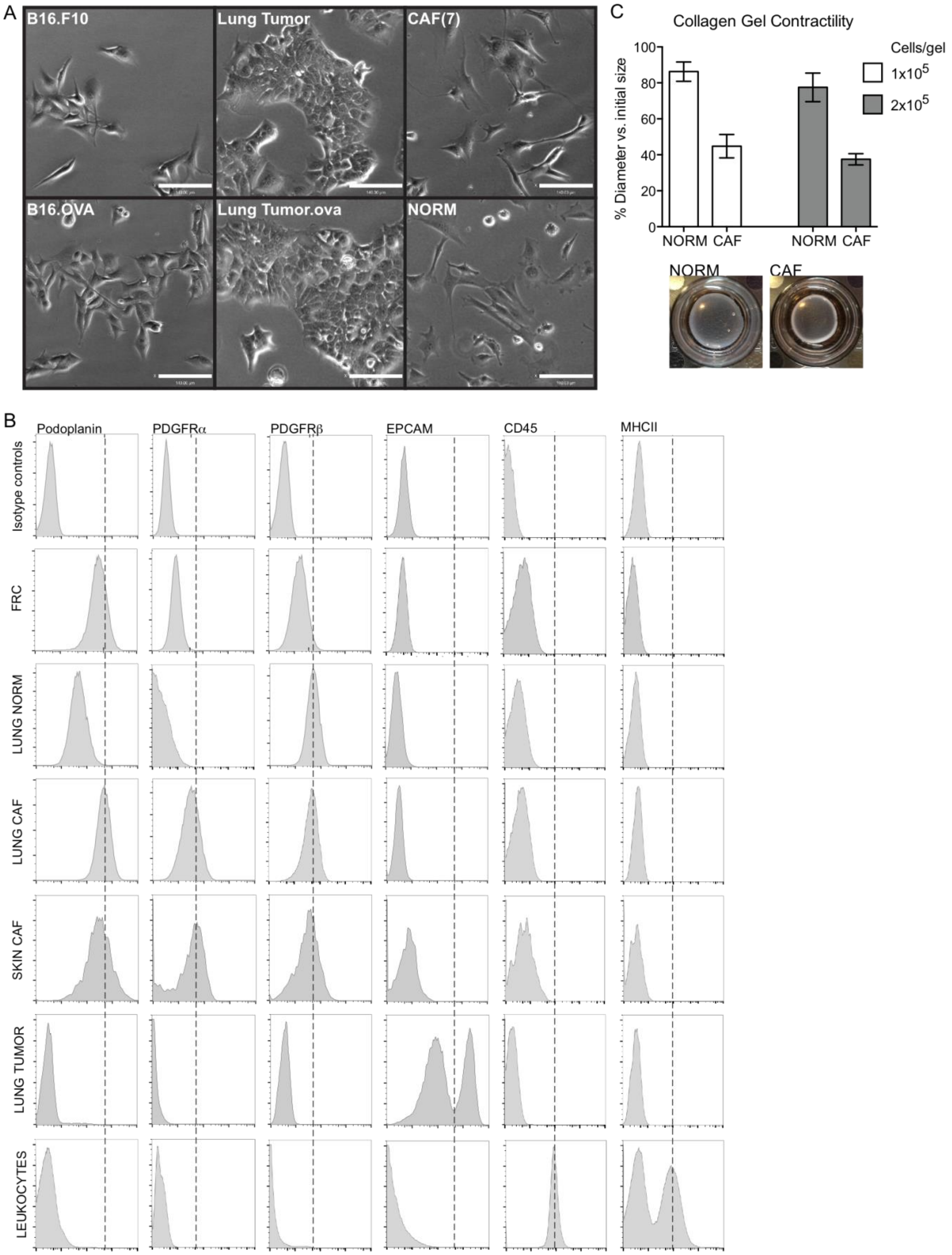

Supplementary figure 1. Tumour cell and fibroblast phenotypes *in vitro*. (**A**) Representative phase contrast images of tumour cells B16.F10, B16.OVA, Lung tumour and Lung tumour.OVA and fibroblasts CAF and NORM in *in vitro* culture. Scale bar, 140μm. (**B**) Flow

cytometry characterization of fibroblasts used (NORM, lung CAF, skin CAF and FRC fibroblasts) to exclude tumour cell and immune cells. Representative profiles for typical CAF markers Podoplanin, PDGFRα and PDGFRβ; epithelial marker EPCAM; and immune markers CD45 and MHC II. (**C**) Fibroblast collagen gel contractility assay displayed as gel diameter as a percentage of its starting diameter.

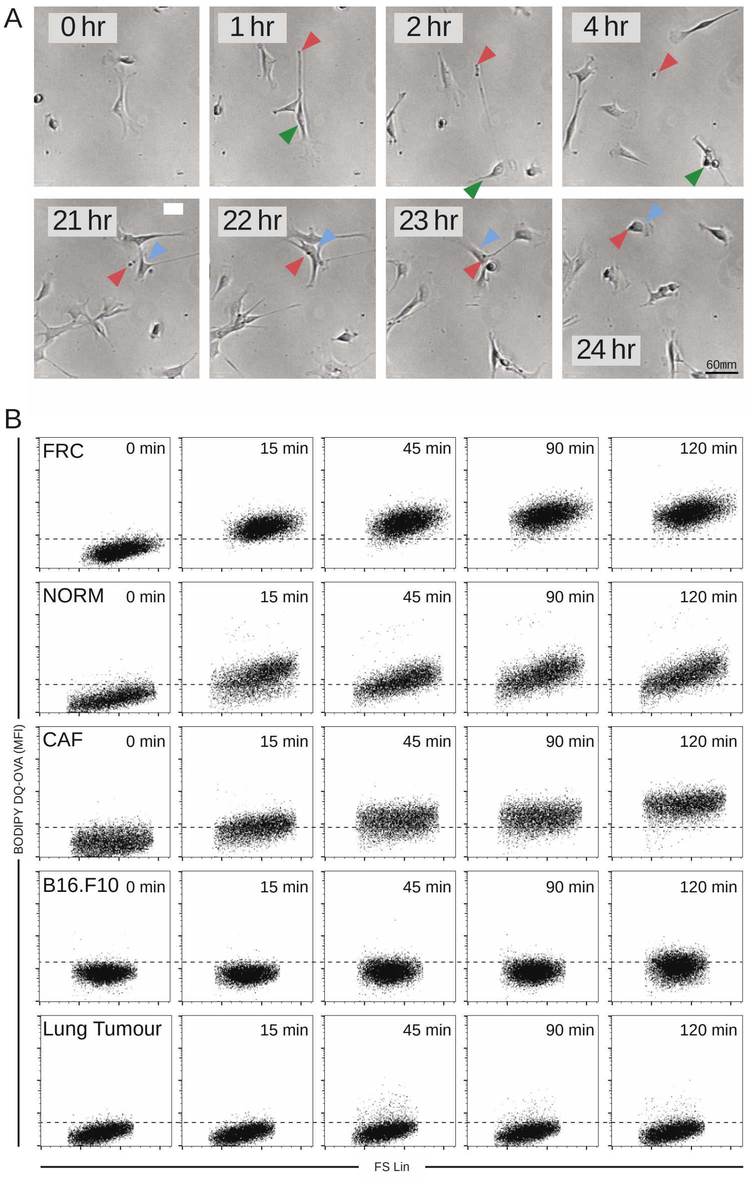

Supplementary figure 2. Ovalbumin processing by fibroblasts and tumour cells *in vitro*. (**A**) Time-lapse phase images showing debris (red arrow) breaking away from a donor cell (green arrow, upper panel 0-4hrs) and its subsequent uptake by an independent acceptor cell (blue arrow, 21-24hrs). Scale bar. 60μm. (**B**) Representative FACS dot plots showing ovalbumin (DQ-OVA) proteolytic degradation over time for FRC, NORM, and CAF fibroblasts and B16.F10 and lung tumour cells.

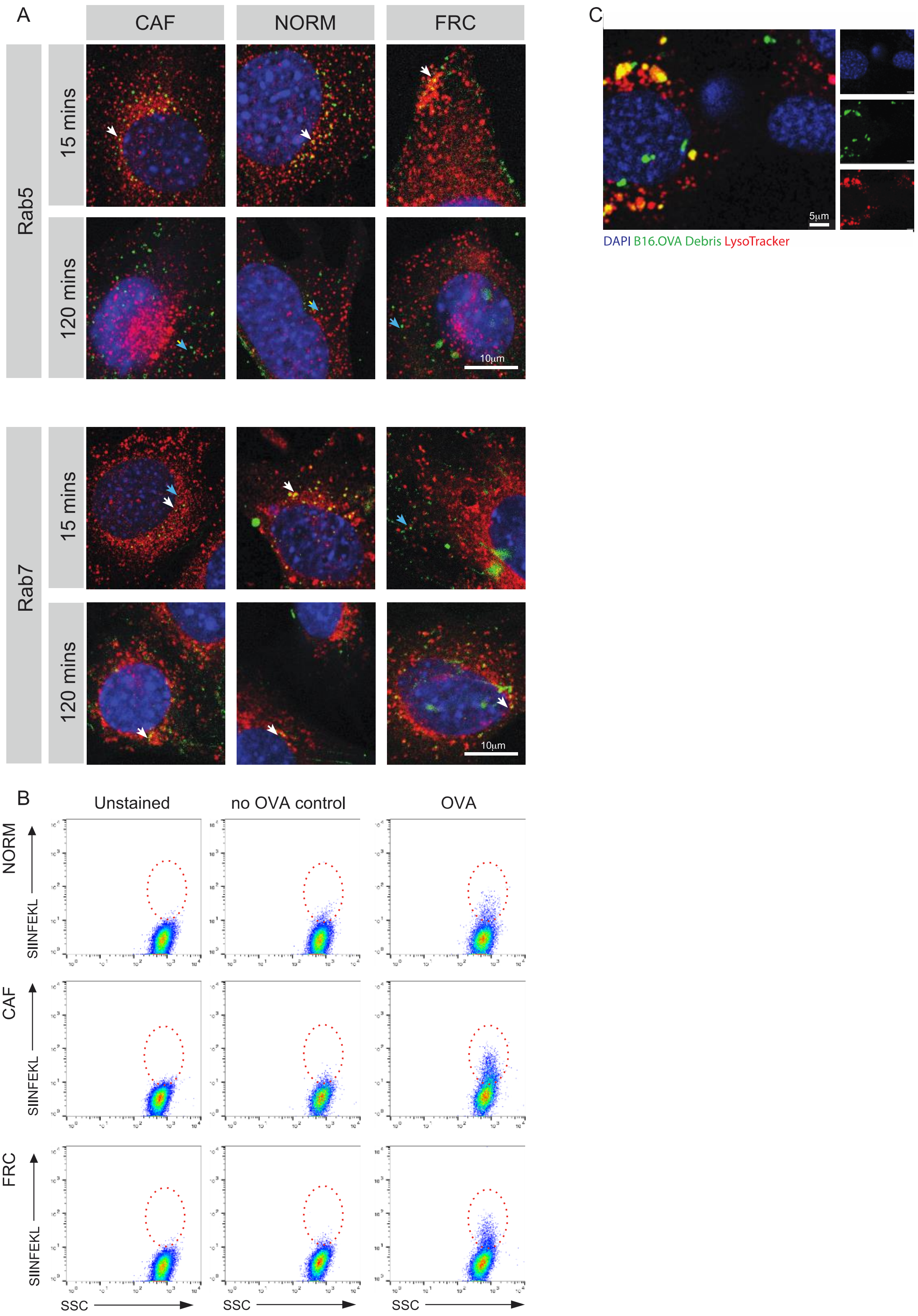

Supplementary figure 3. CAFs exhibit slower processing kinetics than normal fibroblasts. (**A**) Representative micrographs of endosomal compartments (red) showing early endosomes (Rab5), and late endosomes (Rab7) at 15 and 120 minutes post ovalbumin pulse (green). Areas of co-localization (white arrows) and ovalbumin not within labelled compartments (blue arrows) are indicated in each image. Scale bar: 10µm. (**B**) Flow

cytometry plots showing MHC I SIINFEKL staining in FRCs, CAFs and normal fibroblasts 18 hours after pulsing with ovalbumin (unstained control; stained but no OVA pulse control; OVA pulsed). (**C**) Representative micrograph of LysoTracker (red) labelled CAFs containing engulfed B16.OVA tumour debris (green). Nuclei counterstained with DAPI (blue). Scale bar, 5µm. Assays performed in triplicate from 2 experiments (**B**).

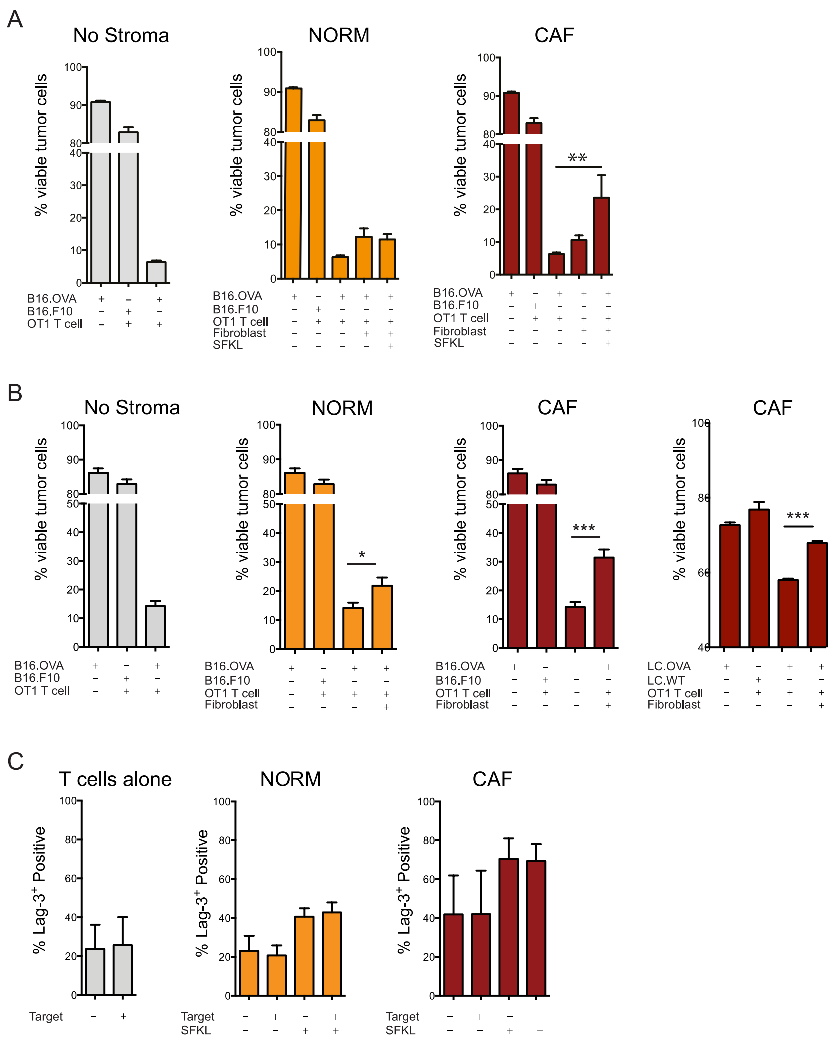

Supplementary figure 4. CAFs reduce CD8^{+} T cell viability in an antigen-specific, death ligand dependent manner. (**A**) T cell capacity to kill tumour cells following fibroblast co-culture (normal fibroblasts, yellow bars; CAFs, red bars) and subsequent transfer to parental (B16.F10) or antigen bearing (B16.OVA) cells. (**B**) T cell capacity to kill tumour cells when control parental (B16.F10) or antigen bearing (B16.OVA) tumour cells were used as source of antigen rather than addition of OVA. Tumour cells were cultured with fibroblasts prior to addition of activated OT-I T cells. Flow cytometry quantification of tumour cell viability in cultured alone or in presence of normal fibroblasts (yellow bars), CAFs (red bars). (**C**) T cell capacity to kill tumour cells when control parental (LC.WT) or antigen bearing (LC.OVA) tumour cells were used as source of antigen rather than addition of OVA. (**D**) Flow cytometry quantification of Lag-3 expression by T cells cultured in presence or absence of fibroblasts, antigen and target cells as indicated. Data shown as mean ± SEM. * $P < 0.05$, ** $P < 0.01$, *** $P < 0.001$ (one-way ANOVA with Tukey's or Dunnett's post hoc analysis). ns, not significant. Comparisons indicated by horizontal lines. Assays performed in duplicate from three independent experiments.

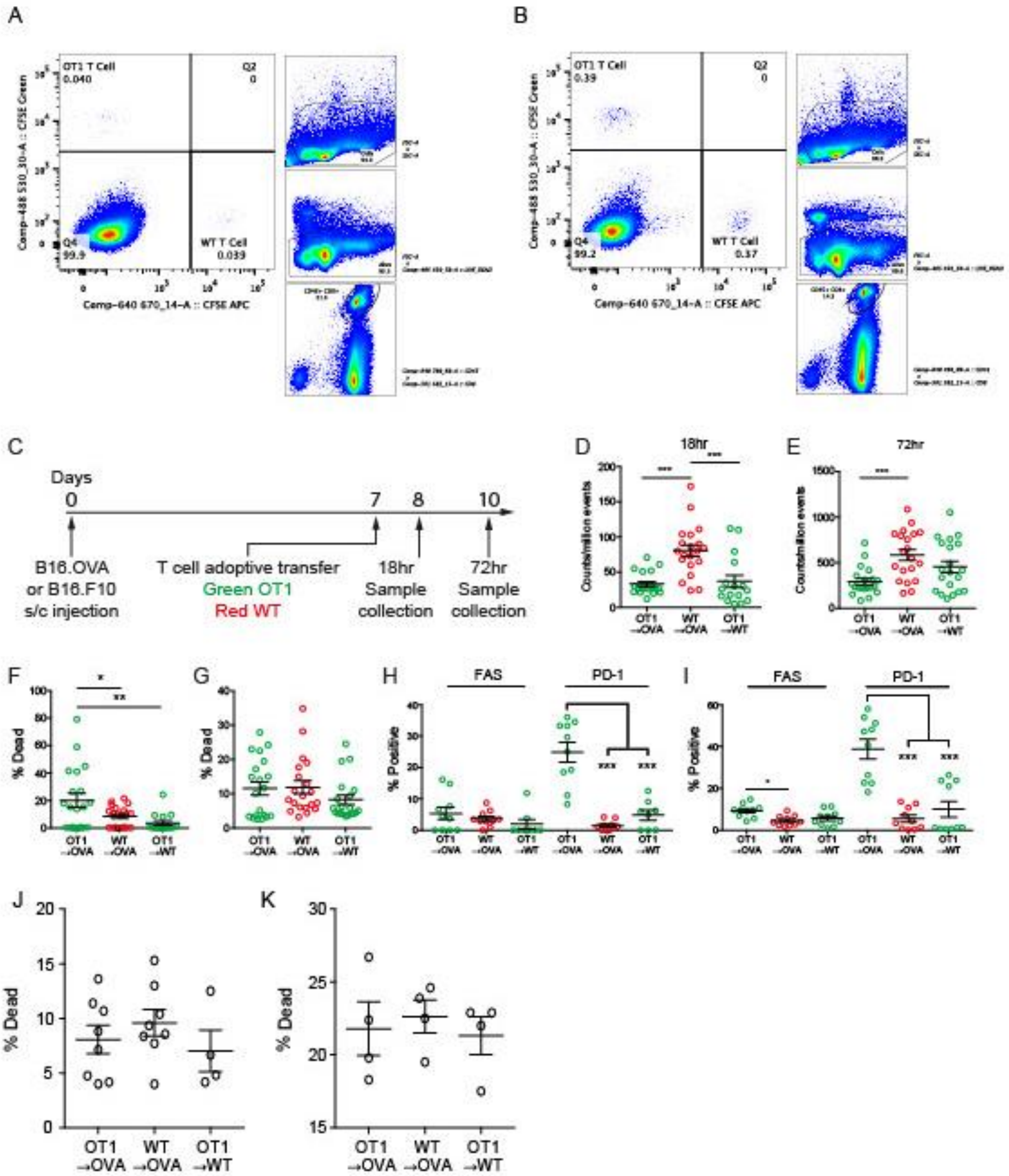

Supplementary figure 5. Antigen-specific deletion of T cells occurs *in vivo*. (**A**-**B**) Gating strategy for recovered T cells after adoptive transfer to tumour bearing mice. Representative plots identifying differentially labelled OT1 T cells (CFSE green) and WT T cells (CFSE APC) from lymph nodes (**A**) and f spleen (**B**) of tumour bearing mice. Main panels depict live-labelled T cells with minor panels illustrating gating ancestry. (**C**) Adoptive transfer experimental design. (**D**) Intratumoural T cell counts/million events at 18hrs and (**E**) 72hrs post transfer. (**F**) Frequency of dead labelled T cells per tumour at 18hrs and (**G**) 72hrs post transfer. (**H**-**I**) FAS and PD-1 expression by live T cells within tumours at (**H**) 18hrs and (**I**) 72hrs post transfer. (**J**-**K**) T cells adoptively transferred show equal viability in the lymph and spleen of recipient mice. CD8+ T cell viability of cells recovered from adoptive transfer of either OT1 or WT T cells in to recipient mice bearing ovalbumin (OVA) or WT B16 tumours

from the (**J**) lymph nodes or (**K**) spleen. Data shown as mean ± SEM. * $P < 0.05$, ** $P < 0.01$, *** $P < 0.001$ (one- way ANOVA with Tukey's post hoc analysis). ns, not significant. Comparisons indicated by horizontal lines. Assays performed in 10 mice (**D**-**G**), 5 mice (**H**, **I**) or 4 mice (**J**, **K**) from two independent experiments. Each point is a individual tumour (**D**-**I**), lymph node (**J**) or spleen (**K**).

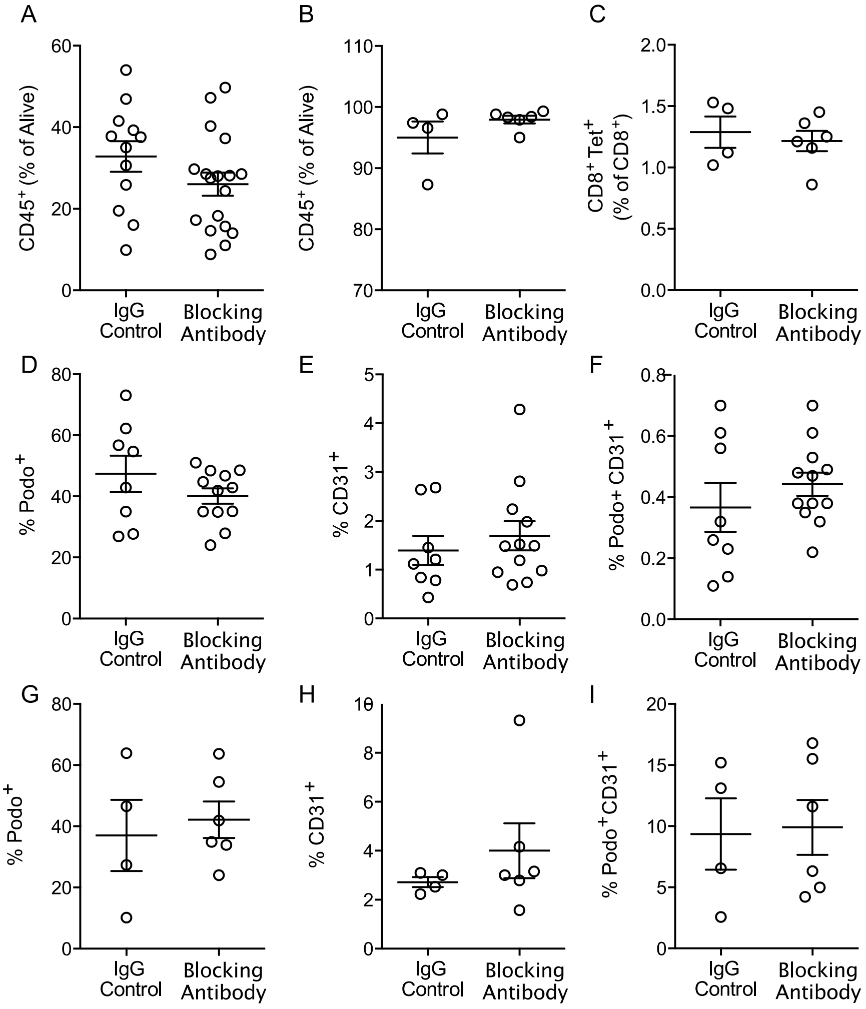

Supplementary figure 6. Blocking antibodies against PD-L2 and FASL have no overall effect on total immune cell number or stromal cell populations in tumours and lymph nodes. (**A**) Total $CD45^+$ immune cell frequency in tumours and (**B**) lymph nodes from mice receiving control antibody or blocking antibodies. (**C**) Tetramer positive CD8 T cells in lymph nodes from mice receiving control antibody or blocking antibodies. (**D**-**F**) Stromal cell populations within tumours from animals receiving either control or blocking antibodies as a percentage of $CD45^-$ cells stained for (**D**) Podoplanin, (**E**) CD31, (**F**) both. (**G**-**I**) Stromal cell populations within lymph nodes from animals receiving either control or blocking antibodies as a percentage of $CD45^-$ cells stained for (**G**) Podoplanin (FRC), (**H**) CD31 (blood vessels) (**I**) both (LECs). (**J**) (**K**) Data shown as mean ± SEM. Significance determined by two-tailed unpaired Student's t-test. Symbols show individual animals.

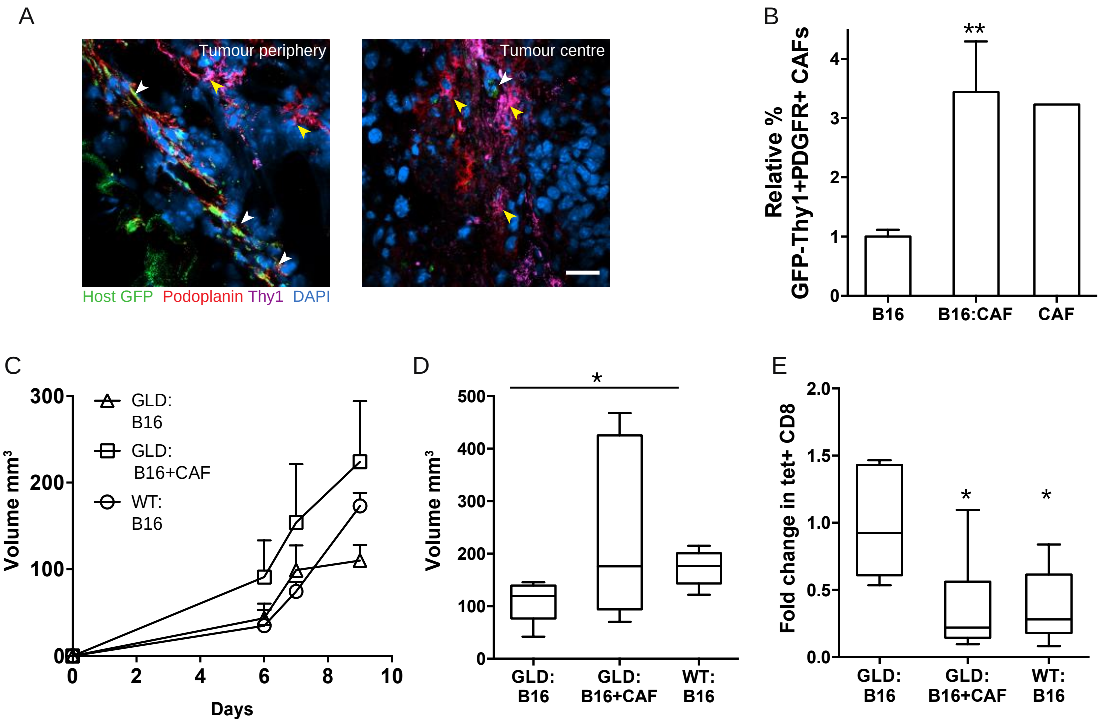

Supplementary figure 7. Impact of deletion of host FASL on tumour volume and antigen specific T cells. (**A**) Representative confocal image confirming presence of injected (GFP^-, yellow arrowheads) $Thy1^+Podoplanin^+$ CAFs within day 9 tumours at the periphery (left) and centre (right) of the lesion. Host-derived cells were detected as GFP^+ (white arrowheads) Scale bar: 20μm. (**B**) Flow cytometry quantification of CFSE-Labelled injected CAFs expressed as relative percentage within the non-immune stromal compartment ($CD45^-GFP^-CFSE^+PDGFR\alpha^+Thy1^+$ cells). ** *P* < 0.01 (one-way ANOVA with Tukey's post hoc analysis). n = 3 individual tumours. (**C**) Growth curves of tumours in FASL-deficient GLD mice. B16.OVA tumour cells were implanted alone (no FASL present in host) or at a 1:1 ratio with CAFs (FASL on CAFs only), and compared with tumours implanted in WT C57 (**D**) Volumes of day 9 tumours. (**E**) Antigen specific T cells in tumours expressed as fold change of Tetramer positive CD8 T cells within the $CD45^+$ compartment. Data shown as mean ± SEM. * *P* < 0.05 (one-way ANOVA with Tukey's post hoc analysis). n = 6 tumours from 2 independent experiments.

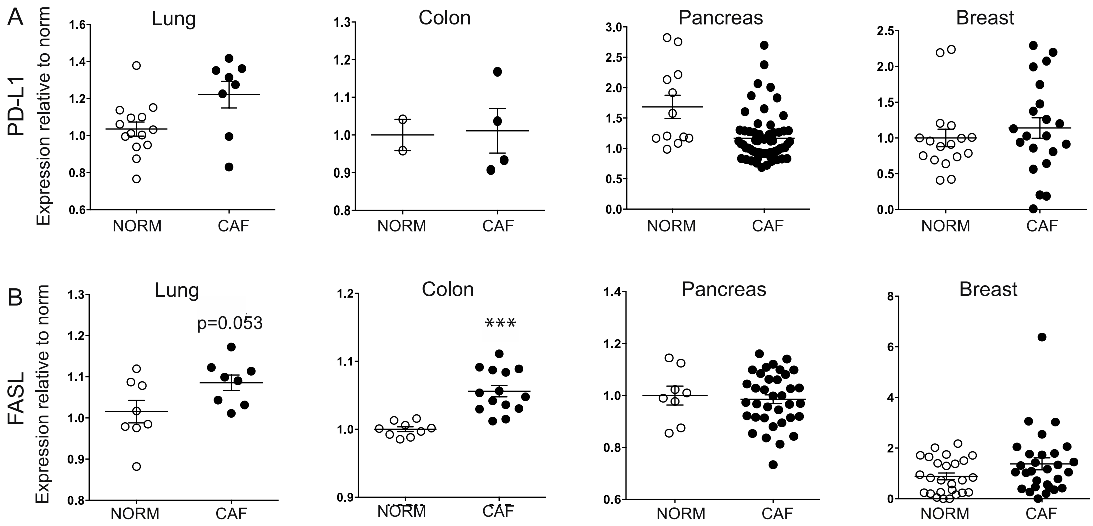

Supplementary figure 8. PD-L1 and FASL expression in human CAFs and normal fibroblasts. (**A**) PD-L1 expression from tumours where data was available (colon, only GSE1257). B) FASL expression. Data shown as mean ± SEM., *** $P < 0.001$. (two-tailed unpaired Student's t-test). Symbols represent individual samples.
