# Supplementary material for: Cancer-associated fibroblasts induce antigen-specific deletion of CD8+ T Cells to protect tumour cells
Source: Nat Commun. 2018 Mar 5;9:948. doi: 10.1038/s41467-018-03347-0 (PMC5838096; doi:10.1038/s41467-018-03347-0)
Supplement: Supplementary file 3 — Description of Additional Supplementary Files [file 41467_2018_3347_MOESM3_ESM.pdf]

Description of Additional Supplementary Files

File Name: Supplementary Movie 1

Description: Confocal live imaging of Cell Tracker labelled CAFs (red) interacting with dead and dying tumour cells (green) presented as a snapshot in Fig 1Di. GFP-labelled tumour cells were killed with puromycin prior to incubation with labelled CAFs and imaged over a period of 24 hours on a Leica DMi8 microscope with environmental chamber. Images were taken every 10 minutes. CAFs can be seen sensing the green cells before prolonged interaction and engulfing cell-derived material.

File Name: Supplementary Movie 2

Description: 3D confocal projection of micrographs in Fig 1Dii and iii illustrating engulfed, intracellular debris (GFP, green) in discrete compartments of the CAF (Cell Tracker, red) at the end of 24 hours. Following live imaging, cells were fixed in 4% PFA for 10 minutes and imaged on a Leica SP5 confocal microscope. Phase contrast of all cells is also depicted.
