# Supplementary material for: Cancer-associated fibroblasts induce antigen-specific deletion of CD8+ T Cells to protect tumour cells
Source: Nat Commun. 2018 Mar 5;9:948. doi: 10.1038/s41467-018-03347-0 (PMC5838096; doi:10.1038/s41467-018-03347-0)
Supplement: Supplementary file 2 — Peer Review File [file 41467_2018_3347_MOESM2_ESM.pdf]

Reviewer #1:

Remarks to the Author:

In their manuscript, Lakins and co-authors reported interesting and convincing data about the involvement of CAFs in immunesuppression, with an active role of CAFs that strictly resemble that of antigen presenting cells. An intriguing role of Fas and PD-L2 in driving death and dysfunction of CD8+ T cells, ultimately leading to tumor cell survival and tumor mass enhancement, has also been suggested and clearly demonstrated. The authors presented a well-done study, in which they clearly demonstrate a novel mechanism of immuneregulation, involving tumor-associated-fibroblasts in the dysfunction of effector T cell. In my opinion, the conclusions provided by the authors are excellently supported by data and give a solid improvement in the current knowledge on tumor immunity.

Reviewer #2:

Remarks to the Author:

The paper by Lakins et al. describes in vitro and in vivo experiments addressing the role of cancer-associated fibroblasts (CAFs) in tolerizing CD8 T cells specific against tumour antigens. The data suggest that CAFs take up antigens (free and cell-bound), process and cross-present them to antigen-specific T cells, and that this results in functional impairment and deletion of the T cells. The interaction involves PD-L2 and FASL expressed by CAFs, and the corresponding ligands PD-1 and Fas, respectively, on T cells. Interventions at the level of these two pathways improve T cell competence.

The project is well thought and focused on burning questions of how tumors avoid destruction by cytotoxic lymphocytes. Several different cell populations of the tumor microenvironment have been proposed to be (co-)responsible for hampering T cell responses. This work is in line with previous studies arguing that stromal cells may be involved. It goes significantly further, as it proposes that CAFs directly tolerize T cells in an antigen-specific and cell-contact dependent manner.

The majority of molecularly and cellularly defined experiments were done in vitro with model antigens and cells, documenting antigen handling by CAFs and their interactions with T cells in vitro. The in vivo experiments are less specific, mainly because these experiments lack CAF-specific interventions. The global targeting of PD-L2 and FASL with antibodies is not sufficient to make the point that CAFs are indeed responsible for the observed effects. It is likely that other and/or additional cell populations are involved.

Without specific in vivo data the principles proposed by the authors is not proven, and the data remain preliminary. Also, their data do not present “first evidence of tumour stroma-directed T cell suppression”.

Minor points:

- Supplemental movies 1 & 2 should have a legend or be described otherwise, the experimental conditions must be documented. It should be explained what these movies show, more than just the very short remarks in the main text.

- Negative control data should be shown, e.g. in the Supplementary Figure 3B

Reviewers' comments:

Reviewer #1:

In their manuscript, Lakins and co-authors reported interesting and convincing data about the involvement of CAFs in immune suppression, with an active role of CAFs that strictly resemble that of antigen presenting cells. An intriguing role of FAS and PD-L2 in driving death and dysfunction of CD8+ T cells, ultimately leading to tumor cell survival and tumor mass enhancement, has also been suggested and clearly demonstrated. The authors presented a well-done study, in which they clearly demonstrate a novel mechanism of immuneregulation, involving tumor-associated-fibroblasts in the dysfunction of effector T cell. In my opinion, the conclusions provided by the authors are excellently supported by data and give a solid improvement in the current knowledge on tumor immunity.

We thank the reviewer for the positive comments with regards to our manuscript.

Reviewer #2:

The paper by Lakins et al. describes in vitro and in vivo experiments addressing the role of cancer-associated fibroblasts (CAFs) in tolerizing CD8 T cells specific against tumour antigens. The data suggest that CAFs take up antigens (free and cell-bound), process and cross-present them to antigen-specific T cells, and that this results in functional impairment and deletion of the T cells. The interaction involves PD-L2 and FASL expressed by CAFs, and the corresponding ligands PD-1 and Fas, respectively, on T cells. Interventions at the level of these two pathways improve T cell competence. The project is well thought and focused on burning questions of how tumors avoid destruction by cytotoxic lymphocytes. Several different cell populations of the tumor microenvironment have been proposed to be (co-)responsible for hampering T cell responses. This work is in line with previous studies arguing that stromal cells may be involved. It goes significantly further, as it proposes that CAFs directly tolerize T cells in an antigen-specific and cell-contact dependent manner.

The majority of molecularly and cellularly defined experiments were done in vitro with model antigens and cells, documenting antigen handling by CAFs and their interactions with T cells in vitro. The in vivo experiments are less specific, mainly because these experiments lack CAF-specific interventions. The global targeting of PD-L2 and FASL with antibodies is not sufficient to make the point that CAFs are indeed responsible for the observed effects. It is likely that other and/or additional cell populations are involved. Without specific in vivo data the principles proposed by the authors is not proven, and the data remain preliminary.

We thank the reviewers for their comments with regards to CAF-driven negative stimulation. In light of this, we obtained *gld/gld* mice homozygous for the *fasl*^{gld} mutation, and thus none of the host tissues were able to express FASL. Following implantation of antigen bearing B16.OVA tumours alone or in 1:1 mix with CAFs into *gld/gld* or wild type C57 mice we observed that B16.OVA tumours in mutant mice (where no FASL was present) were smaller than mixed tumours, or B16.OVA in C57 mice (with additional populations expressing FASL). This corresponded with enhanced numbers of antigen-specific tetramer positive CD8 T cells in the mice lacking FASL compared with the mixed tumours or tumours in WT mice. T cells from mixed tumours in mutant mice were not significantly different from WT mice in which FASL effects on intratumoural T cells could potentially come from other sources. Data is presented in Figure S7 and discussed in text on page 4 lines 22-31. Together, these indicate that in the absence of any host-derived FASL, CAFs can support reduced CD8+ T cells consistent with results observed in the systemic blocking studies.

Also, their data do not present "first evidence of tumour stroma-directed T cell suppression".

Although there is plenty of evidence to suggest a role for CAFs in suppression of immune cell function through secreted factors, we believe this to be the first example of direct antigen-specific ligand-receptor mediated interactions between CAFs and T cells within the tumour microenvironment having not been able to find similar published reports. We have modified the text to present a clearer, more specific statement on page 1 line 22.

Minor points:
Supplemental movies 1 & 2 should have a legend or be described otherwise, the experimental conditions must be documented. It should be explained what these movies show, more than just the very short remarks in the main text.

We thank the reviewer for this comment. Detailed legends can be found in Supplementary Materials with specific references to these made in text page 2, lines 18 – 22).

Negative control data should be shown, e.g. in the Supplementary Figure 3B
We appreciate this being pointed out, and representative control plots including 'unstained negative controls' and 'no OVA but stained negative controls' have been added to Supplementary Figure 3B.

Reviewer #2 (Remarks to the Author):

In the revised paper, new experiments are described on page 4 with gld/gld mice known to be FASL deficient. The authors refer to Supplementary Fig. 6A and B, which however show data that are completely unrelated, likely a mistake. Probably the authors mean Supplementary Fig. 7A and B, suggesting that B16 tumors reach low and similar sizes in GLD hosts and in WT hosts. Eventually larger tumors size is observed in GLD hosts when B16 are injected together with CAFs, arguing that FASL of the CAFs is responsible for less CD8 T cells, similar to WT hosts. Are the differences in tumor sizes significant? Unfortunately, the data does not show the kinetics of tumor growth, only one time point (day 9) is shown. It should also be determined whether the injected CAFs actually survived and made up a significant part of the tumor stroma. Moreover, several controls are missing, particularly the combined injection of B16 + FASL deficient CAFs. Further controls should be added to elucidate why tumor sizes are similar in GLD hosts and in WT hosts, despite that they differ in FASL. The model is not clean since, the mice may differ in further traits as a consequence of the genotype difference.

Please find responses to additional points raised by reviewer two.

In the revised paper, new experiments are described on page 4 with gld/gld mice known to be FASL deficient. The authors refer to Supplementary Fig. 6A and B, which however show data that are completely unrelated, likely a mistake. Probably the authors mean Supplementary Fig. 7A and B, suggesting that B16 tumors reach low and similar sizes in GLD hosts and in WT hosts.

We thank the reviewer for highlighting the typographical error. This has now been altered in the text, correctly referring to Supplementary Figure 7.

Fig. 7A and B, eventually larger tumors size is observed in GLD hosts when B16 are injected together with CAFs, arguing that FASL of the CAFs is responsible for less CD8 T cells, similar to WT hosts. Are the differences in tumor sizes significant?

The differences between B16 in GLD mice and B16 in WT mice has a P<0.05, the asterisk moved to a lower layer during figure preparation. We thank the reviewer for spotting this and it has now been moved back to the front of the graph.

Unfortunately, the data does not show the kinetics of tumor growth, only one time point (day 9) is shown.

We chose to depict final volumes to be consistent other data presented in the manuscript. However, we have now added the matching growth curve data to Supplementary Figure 7, which illustrates the stasis in growth of B16 in the FASL-deficient GLD mice starting from day 6 in comparison to WT animals where tumour growth continues to takes off.

It should also be determined whether the injected CAFs actually survived and made up a significant part of the tumor stroma.

We have added data to Supplementary Figure 7 which illustrates the presence of injected CAFs at the time of tumour harvest. Confocal images in Supplementary Figure 7A depict both endogenous host-derived podoplanin+Thy1+GFP+ and (white arrows) and transplanted podoplanin+Thy1+GFP- (yellow arrows) CAFs at the periphery, and predominantly donated podoplanin+Thy1+GFP- cells CAFs at the centre of the tumour. Data were also quantified in 7B.

Several controls are missing, particularly the combined injection of B16 + FASL deficient CAFs.

Since we have shown the role of CAF-expressed FASL in multiple models, the generation and implantation of FASL-deficient CAFs into the cohort of GLD mice that were made available to us would not add anything to substantially strengthen the story or impact conclusions; this condition is naturally included within the B16-GLD group in the form of endogenous CAFs with non-functional FASL.

We have shown in vitro that FASL on CAFs can mediate reduced antigen specific T cell numbers and thus impaired target tumour cell killing. To confirm this, we blocked FASL function specifically on CAFs in vitro; when CAF-FASL was neutralized T cell death was prevented and antigen specific killing capacity of tumour cells reinstated – thus blocking CAF FASL supports more antigen specific T cells and smaller tumours. This was reinforced in two in vivo systems namely the systemic blocking studies and the use of GLD mice. With systemic blocking of FASL function, we detected more antigen specific T cells and smaller tumours. In GLD tumour-bearing mice containing endogenous CAFs with non-functional FASL (equivalent to implanting FASL-deficient CAFs, or pre-blocking CAFs specifically), antigen specific T cell numbers were higher and tumours smaller, consistent with other methods tested.

Further controls should be added to elucidate why tumor sizes are similar in GLD hosts and in WT hosts, despite that they differ in FASL. The model is not clean since, the mice may differ in further traits as a consequence of the genotype difference.

The volumes of B16 in WT and GLD are significantly different, and the time course data illustrates a stasis in tumour growth specifically in GLD mice from day 6. This mouse model was chosen as relevant to support to previous blocking studies which were less specific in nature. We have used this system to detect a role for FASL, one of the suppressive ligands present within the tumour stroma microenvironment. FASL is not the sole suppressive intermediate and hence, also in GLD mice, other contributing suppressive mechanisms beyond FASL (which we have not explored in the context of this manuscript) may exist. Nevertheless, tumours in GLD mice are smaller and contain more antigen specific T cells than either those co-implanted with CAFs or in C57 mice, entirely consistent with our in vitro and systemic blocking assays.

We are aware that beyond 20 weeks of age, mice may exhibit some autoimmune symptoms such as enlarged spleens, lymph nodes and kidney dysfunction. Mice used in this study were young (8 weeks) for a period of 9 days, at which time no evidence of lymph node enlargement or associated abnormalities were detected. If we were to instead utilize a FASL-/- mouse, indeed there would be a more severe systemic phenotype than the GLD with an extracellular mutation which blocks interaction with the FAS (more closely mirroring the FASL neutralization approach).

Reviewer #2 (Remarks to the Author):

The revisions were well done, by taking my questions and suggestions sufficiently into account. I have no further comments.
